# Strengthening surgical outcomes through nursing education: an evidence-based training model in Laos

**DOI:** 10.3389/fpubh.2026.1756562

**Published:** 2026-01-27

**Authors:** Pei Yan, Chunxiao Wang, Lamngeun Singphandy, Yanru Shi, Xiaohua Chen, Yanfang Li, Qiao Cheng, Jin Yang, Xiaoyu Zhou, Xuehui Hu

**Affiliations:** 1 Xi Jing Hospital, The Fourth Military Medical University, Xi’an, China; 2 103 Military Central Hospital, Vientiane, Laos

**Keywords:** Laos, nursing education, patient safety, perioperative care, training model

## Abstract

**Background:**

The quality of perioperative care is closely linked to postoperative recovery. The level of care in low- and middle-income countries hinders the pace and quality of postoperative recovery. It is essential to understand the perioperative situation and needs of local hospitals to target the development of localized professional training programs, thereby enhancing the professionalism of nursing staff in providing high-quality care to patients.

**Objective:**

To study issues related to the quality of perioperative nursing in hospitals in Lao and improve the threshold of holistic care.

**Methods:**

Qualitative and quantitative studies were used to design the training program. In total, 25 nurses from a hospital were selected between December 2024 and May 2025. A survey was conducted to understand issues with the targeted construction of training programs and their implementation, evaluation, and continuous supervision. A comparative analysis of operation scores and the competence level of each nurse before and after the training was conducted to evaluate the training’s effectiveness.

**Results:**

A total of 11 aspects were identified using the Knowledge, Attitude, and Practice model. In total, 30 theoretical and 12 operational training topics were designed, and a training strategy was developed to enhance the quality of perioperative care, based on preoperative, intraoperative, and postoperative data. A STEP-CARE model was designed to ensure overall quality improvement and training effects. Hand hygiene compliance, accuracy, and performance were also improved.

**Conclusion:**

The study recommends using knowledge, attitudes, and actions as starting points for continuous quality improvement. Training qualified professionals, improving institutional processes, strengthening disciplinary collaboration, and improving facilities are imperative to enhance the overall outcome.

## 1 Introduction

Perioperative care is the link between the preoperative, intraoperative, and postoperative periods. The quality of perioperative care is directly related to surgical safety, postoperative recovery, efficiency of medical resource use, and overall satisfaction with medical services (1). Systematic literature review shows that surgical incidents accounted for the highest proportion (39.6%) of adverse events in hospitals (2). An analysis of medical disputes in Korea further reveals that surgical cases account for the highest proportion of claims (35.1%), with the trend showing a gradual upward trend (3). Similarly, optimizing the care management model after pancreaticoduodenectomy surgery can reduce the incidence of postoperative complications from 34.21 to 15.79%, initial ventilation time by 10.9 h, and length of postoperative hospital stay by approximately 82 h (4), optimizing resource allocation and healthcare costs. Hypothermia is a common and preventable complication in surgical patients, associated with serious adverse outcomes such as increased bleeding, surgical site infections (SSIs), cardiovascular events, and prolonged hospital stays, the knowledge and practice of perioperative nurses in temperature management are crucial for ensuring patient safety (5). These findings indicate that adverse surgical events and outcomes are global concerns. Improving the quality of perioperative care not only helps deliver robust clinical outcomes but also boosts the overall efficacy of healthcare organizations, which is at the core of managing a modern healthcare system.

In 2009, the World Health Organization (WHO) developed guidelines to identify practices that could ensure surgical safety worldwide (6). The implementation of the surgical safety checklist facilitates the early identification and management of potential complications, thereby improving patient surgical outcomes (7). Similarly, instrument errors were reduced from 48.6 to 60.7% after the use of preoperative checklists (8).

The imperative for high-quality perioperative patient safety management has become a global healthcare priority, with nations worldwide developing strategies tailored to their local contexts. For instance, a major international study by Haynes et al. confirmed that systematic surgical safety management significantly reduces mortality and postoperative complications (9). Quality improvement interventions implemented in a pediatric intensive care unit successfully reduced the Central Line-Associated Bloodstream Infection rate by 74% and ultimately achieved a 6-month CLABSI-free period (10). Therefore, perioperative patient safety management has become a patient safety initiative aimed at reducing the risks associated with surgery (11–13). Central to these safety initiatives is the role of high-quality operating room (OR) nursing. Practices such as strict aseptic technique, meticulous skin preparation, and proper instrument management are critical for preventing surgical site infections (SSIs)—common and serious complications that lead to prolonged hospital stays, increased costs, and higher mortality (7). Beyond reducing clinical risks, the quality of perioperative care directly impacts patient experience. Indeed, high-quality care is a key driver of patient satisfaction, a crucial metric for evaluating healthcare services (14). The positive impact of continuous quality improvement is further exemplified in advanced procedures like da Vinci robot-assisted surgery, where enhanced perioperative care leads to significantly better clinical outcomes (15).

The Lao People’s Democratic Republic (PDR), with a population of 7.5 million in Southeast Asia (16), is a lower–middle-income country in the WHO Western Pacific Region (17). The team involved in this study has been helping the hospitals in Lao to improve their overall care for five consecutive years. However, there are some challenges in perioperative patient safety management and care, including a lack of resources, weak infrastructure, and insufficient human resources. Daily work, based on experiential teaching, lacks overall system and standardization in nursing management. Reviewing the literature, we find no research on perioperative care quality improvement strategies in Laos hospitals. This study bridges the gap by combining local nursing needs based on the status quo survey. With the aim of improving the quality of perioperative care and patient safety, and under the guidance of the Knowledge, Attitude, and Practice (KAP) model, a continuous quality improvement model is constructed through the mastery of theoretical knowledge and changes in beliefs and behaviors to promote continuous improvement of the quality of perioperative care for patients. Nurse training strategies in this study were designed by the authors according the assistant experience in 5 years. The hospital supported by the author’s unit is the largest military hospital in Laos. On the one hand, the clinical application of this training model will improve the overall level of local surgical care by enhancing the theoretical and operational skills of the surgical nursing staff. On the other hand, raise health awareness among patients and families and promote the health of the local population.

## 2 Materials and methods

### 2.1 Participant recruitment

Between December 2024 and May 2025, a pilot study using a single-group pretest-posttest design and purposive sampling strategy was conducted at a large tertiary hospital in Vientiane, Lao PDR. The participants were nurses (n = 25) working in a surgical unit or operating theater in the country. To ensure comprehensive representation, one nurse was recruited from each of the 25 surgical departments. Eligible nurses were those who had required a valid nursing license and completed training (3 months) during the “Perioperative Nursing Quality Improvement Training Course” from January to April 2025. Exclusion criteria included unwillingness to participate or an anticipated inability to complete the program. Initially, 25 nurses were enrolled and completed the training.

### 2.2 Study design

This study employed a mixed-methods approach and was conducted from December 2024 to February 2025. The primary objective was to systematically evaluate the existing nursing workflow, identify nursing problems and areas for capability enhancement, and thereby construct a scientific, evidence-based training framework. The process was carried out in three steps. First, through interviews, on-site observations, and standardized assessments, we systematically identified the key issues and learning needs within perioperative nursing care (covering preoperative, intraoperative, and postoperative phases) concerning the work environment, equipment conditions, personnel skills, rules and regulations, and workflows. Second, based on these identified issues and the field investigation, we defined the training focus on five core nursing competencies: preoperative assessment, intraoperative collaboration, postoperative monitoring, emergency management, and infection control. Third, guided by the Knowledge-Attitude-Practice (KAP) model, we designed targeted training content, objectives, and implementation strategies to ensure that nursing staff master the relevant knowledge and to facilitate the effective translation of theoretical knowledge into clinical practice.

### 2.3 Ethical considerations

The Ethics Committee of the Institutional Review Board of the First Affiliated Hospital of the Fourth Military Medical University of the People’s Liberation Army (No. KY20254199-1) discussed and developed training programs in collaboration with the 103 Military Central Hospital in Vientiane, Lao. Research studies on humans (individuals, samples, or data) are to be conducted in accordance with the principles outlined in the Declaration of Helsinki. The participants were fully informed of the study’s purpose and signed an informed consent form before participating in the study. All data were collected and analyzed anonymously, and participants were allowed to discontinue participation at any time, even if they had previously agreed to continue. Once the students completed all the training and assessment requirements, they were awarded a Certificate of Training Competence. The top three performers in a single practice were also awarded the title of “Professional Operator.”

### 2.4 Building a training system

#### 2.4.1 KAP model

**Figure 1:**
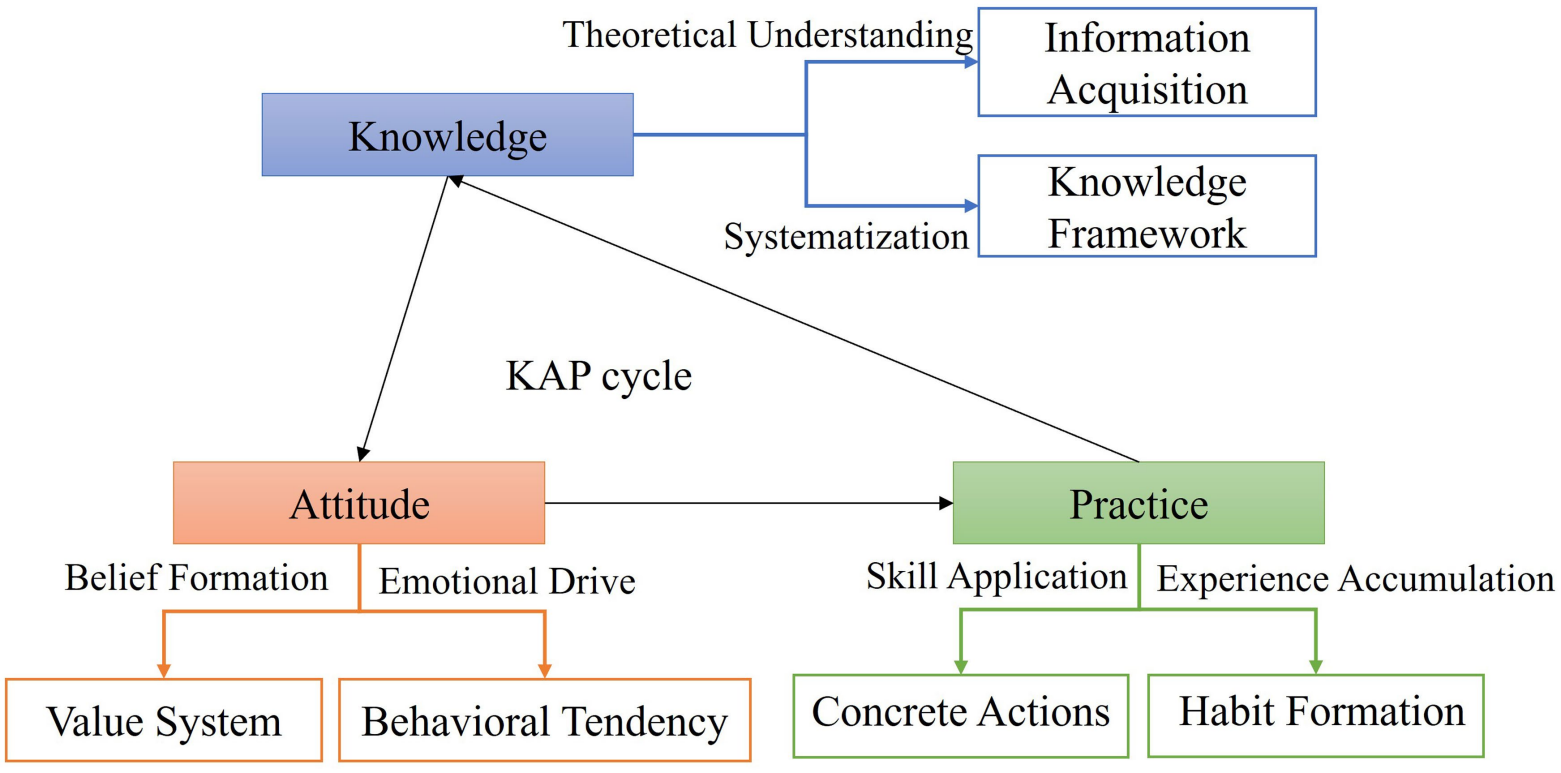
KAP cycle model.

The KAP model is a classic framework used to assess and improve health behaviors in target populations. This model facilitates systematic change in people’s behavior by enhancing knowledge, refining attitudes, and promoting practical application (18). The extended concept of this study, based on the KAP model, is illustrated in Figure 1.

#### 2.4.2 KAP model evaluation

To ensure the scientific rigor of the research instrument, we assessed its reliability and validity after the evaluation form was developed. The instrument’s validity was evaluated using content validity. After finalizing the questionnaire and assessment checklist, we invited 10 domain experts (including 5 Chinese and 5 Laos nursing experts) to conduct a review. By calculating the Content Validity Ratio (CVR), we ensured the questionnaire’s content was comprehensive and unambiguous, and that the observation checklist covered all key operational steps. The instrument’s reliability was assessed in three aspects. First, Cronbach’s alpha coefficient was used to evaluate the internal consistency of the questionnaire, with a standard of *α* > 0.7. Second, to assess the questionnaire’s stability, 30 experts were invited to re-evaluate it after a two-week interval. Third, to ensure the objectivity of the performance observations, two researchers independently observed the same nurse’s procedure, and the Kappa coefficient (with a standard of *κ* > 0.6) was used to assess the consistency of the scoring.

### 2.5 Training framework design principles

Prioritizing the promotion of low-cost, easy-to-use quality management tools and training methods based on existing equipment and human resources in Laos is essential. Training content should be co-developed with local nursing leaders to ensure cultural compatibility and acceptance. Training focuses on real-life cases and scenarios, reinforcing operational skills rather than on theoretical indoctrination.

### 2.6 Training methods and formats

#### 2.6.1 Knowledge aspect

Knowledge was primarily delivered through theoretical lectures combined with operational demonstrations that were parts of short- and long-term training programs. Short-term topics were implemented in conjunction with the needs and objectives of each batch of aid hospitals. They were a combination of offline theory and online learning. Long-term training was based on the long-term nursing work plan for the Chinese Army Aid to the Older Adult Expert Group, which is regularly updated in conjunction with the AORN (Association of Operating Room Nurses) and CORN (Chinese Operating Room Nurses) nursing practice guidelines.

#### 2.6.2 Attitudinal aspect

Scenario simulation and role-playing were used to establish perioperative adverse event cases and simulate scenarios involving adverse event management and nurse–patient communication. Nurses with the highest scores in skills assessment were awarded the honorary title of “Professional Operator” and certificates of encouragement.

#### 2.6.3 Clinical practice

Nurses were guided to master the standardized nursing operation process through skill competition and group operation practices in the form of a workshop. Professional operators guided nurses in the clinic to complete relevant operations and clinical applications of theoretical knowledge, continuously improving nursing quality through point-to-point and supervisory feedback.

### 2.7 Assessment and evaluation system

#### 2.7.1 Knowledge aspect

Mastery of theoretical knowledge was assessed through a theoretical examination, and mastery of operational knowledge was evaluated using a standard practical assessment. All the assessment criteria were based on local standards and adapted to the latest international requirements.

#### 2.7.2 Attitudinal aspect

Quality supervision was conducted at regular intervals in the clinic by support specialists, with professional operators summarizing and providing feedback on clinical performance (e.g., preoperative preparation and postoperative handover) during the morning handover.

#### 2.7.3 Clinical practice

We evaluated the effectiveness of the training via monitoring indicators. Nursing performance was evaluated across seven key domains using a five-point Likert scale: Aseptic Technique, Emergency Response, Preoperative Assessment, Pain Management, Infection Control, Nurse–Patient Communication and Postoperative Monitoring. We evaluated the proficiency of four clinical procedures—aseptic technique, wound management, indwelling urinary catheterization and cardiopulmonary resuscitation—before and after a targeted intervention using a percentage-based scoring system aligned with the CORN operational assessment steps and process standards.

### 2.8 Resource security and continuous improvement

A multidisciplinary teaching team comprising nurses, surgeons, and anesthetists was established. Course content was updated annually in accordance with the latest guidelines (e.g., ERAS rapid recovery concept, infection control standards, and theater nursing practice guidelines). A training record management system was established to track nurses’ career development. The results of the training evaluation were linked to title progression and performance to increase the motivation for participation.

### 2.9 Data analysis

Statistical analyses were performed using IBM SPSS Statistics version 27. Normally distributed quantitative data were presented as $\bar{x}\;$± s (SD). Paired t-test were used to assess the differences in students’ scores before and after training. Changes in hand hygiene compliance, accuracy, and performance before and after training were presented in histograms. Radar charts were used to analyze changes in students’ skills. A two-tailed significance level of *α* = 0.05 was adopted for all statistical tests.

## 3 Results

The total number of participants was 25 nurses from 23 units. Of all, 68.0% were aged below 30 years, 96.0% had a high school or secondary school education, and 76.0% had less than 5 years of experience as a healthcare provider. None of the patients had received prior training. The respondents’ general characteristics are listed in Table 1.

**Table 1:** Characteristic of trainees (N = 25).

Charactieristic	Variable	Frequency (n)	Percentage (%)
Gender	Men	10	40
	Women	15	60
Age	26–30	17	68
	30–34	8	32
Education	Junior high school	2	8
	Senior high school	22	88
	University	1	4
Department	Internal medicine	5	20
	Surgery	15	60
	Outpatient/emergency	2	8
	Others	3	12
Clinical experience (years)	<1	8	32
	1–5 5–10	11 6	44 24

The survey process, combined with the KAP model, was used to collate the relevant and existing issues. The findings were then dissected into 10 target directions and 11 aspects, as shown in Table 2.

**Table 2:** Results of the status survey based on the KAP model.

Dimension	Observation	Situations
Knowledge	(1) Fill the theoretical training gap. (2) Acquire core knowledge and skills in perioperative care. (3) Solve the problem of “not knowing how to do it.”	(1) Relying on experience, rather than standardized processes for nursing practice. (2) Lack of systematic training in infection prevention and control (e.g., hand hygiene, infection control, instrument sterilization). (3) Inadequate ability to recognize postoperative complications (e.g., pressure ulcers, early signs of deep vein thrombosis). (4) Inadequate core systems (e.g., no safety checklist system or item counting system).
Attitude	(4) Establish a safety culture identity. (5) Eliminate negative attitudes (e.g., not enough resources to improve). (6) Strengthen the team’s focus on standardized practice. (7) Address “reluctance” to do the job.	(5) The belief that it is difficult for existing conditions to meet international standards. (6) Lack of postoperative infection prevention and control measures (e.g., hand hygiene, urinary tract infections, wound infections, etc.). (7) Conflict between traditional customs and modern medical norms and reliance on empirical teaching
Practice	(8) Encourage sustainable behavior. (9) Translate knowledge and attitudes into actionable and monitorable daily behaviors. (10) Solve the “cannot do” problem.	(8) Simplified operation owing to a lack of equipment. (9) Lack of postoperative care processes (lack of postoperative care standard operating procedures and discharge follow-up mechanisms) (10) Incomplete data records, mostly paper-based, making it challenging to track improvements (11) Core regimen not implemented as required

In light of the status problems identified in this study, the course content was defined from the preoperative, intraoperative, and postoperative perspectives, and teaching methods, evaluation methods, and quality objectives were clarified. Experts in perioperative nursing were invited to review the courses, resulting in the review of 29 courses (Table 3).

**Table 3:** Development of a training program to improve the quality of perioperative care.

Dimensions	Three items	Knowledge	Attitude	Practice
Program content	Preoperative	Guidelines for fasting and water abstinence Guidelines for the prevention and control of surgical site infections	(1) Patient anxiety assessment (2) Informed consent communication (3) Precautions	(1) Surgical safety review (2) Intravenous fluids (3) Skin preparation (4) Pressure ulcer prevention
	Intraoperative	Use of the surgical safety checklist Monitoring depth of anesthesia Monitoring intraoperative hypothermia	(4) Team resource management (5) Leadership development (6) Intraoperative safety risks	(5) Safe use of high-frequency electro-acupuncture (6) Recognition and prevention of hypothermia (7) Patient positioning (8) Use of lumpectomy instruments (9) Intraoperative pressure ulcer prevention (10) Aseptic technique (11) Use of isolation techniques
	Postoperative	Pain assessment method VTE risk assessment Stress injury risk assessment	(7) Data protection practices (8) Adverse event reporting culture (9) Complication risk assessment (10) Adverse event risk assessment	(12) Wound care (13) Equipment operation and training (14) Drainage, urinary catheter, and other tube care (15) Psychological care (16) Pain management
Teaching methods	Preoperative	Theoretical lectures Self-study	(11) Role play (12) Standardized patient interaction	(17) Simulation model exercise (18) Operating theater field exercise
	Intraoperative	Scenario simulation Group discussion	(13) Multidisciplinary team simulation exercise (14) Stress scenario testing (15) Theoretical lectures	(19) Operation workshop (20) Simulation of controlling bleeding
	Postoperative	Case study Demonstration Theory and practice	(16) Quality of care assessment (17) Theory learning	(21) Wound care simulation (22) Patient education role play
Assessment methods	Preoperative	Online theory test Case analysis report	(18) Communication skills assessment (19) Patient satisfaction (20) Simulation scenario evaluation	(23) Checklist implementation (24) Integrity scoring (25) Operational video playback assessment
	Intraoperative	Emergency theory test Equipment awareness test	(21) Teamwork assessment (22) Crisis management protocol (23) Intraoperative supervision	(26) Evaluation of operating specifications (27) Recording of errors in the use of equipment
	Postoperative	Pain assessment accuracy test Risk assessment simulation test	(24) Testing privacy scenarios (25) Reporting system usage statistics (26) Case sharing	(28) Operational standardization score (29) Correct patient compliance rate

The systematic training, team empowerment, equipment adaptation, practice standardization, community-driven, adaptive, resilient, and evaluation (STEP-CARE) model was adopted to implement the course training program based on the construction of a training system. This was accomplished in three stages.

The first stage involved cognitive input, learning, and localization of international guidelines (e.g., using the WHO safety checklist), combining theoretical knowledge with case sharing and scenario simulation.

The second stage involved cultural internalization; for example, conducting science outreach and promotional activities on World Hand Hygiene Day, and observing the effect of direct visualization using an adenosine triphosphate protein fluorescence detector. This helped patients and their families understand the importance of hand hygiene and increase health awareness through a visual experience.

**Figure 2:**
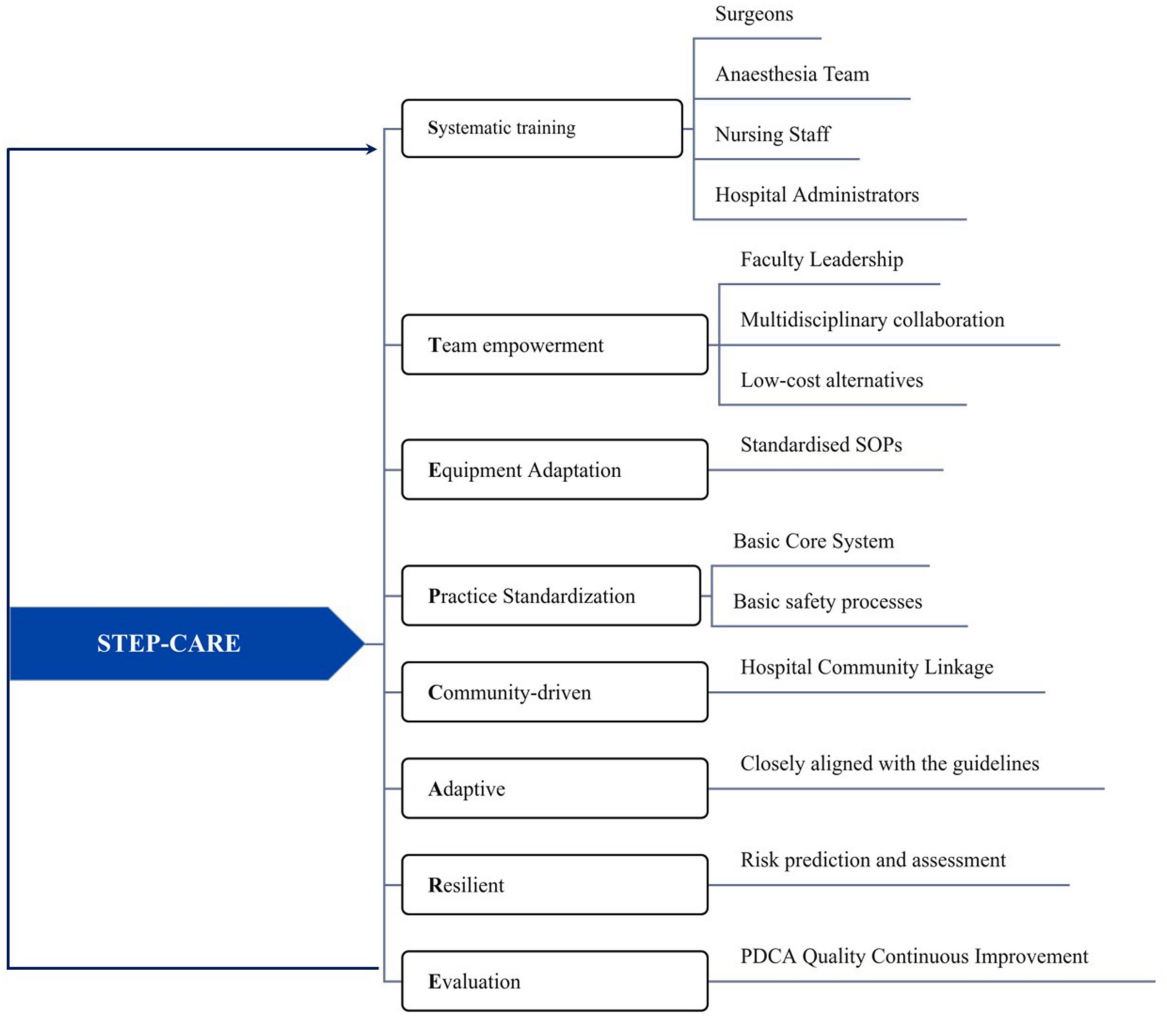
STEP-CARE training model.

In the third stage, skills consolidation and nursing skills were continuously improved through various modes, including simulation exercises, skills assessments, and workshops, utilizing the Plan-Do-Check-Act cycle. In this stage, the quality of training was also improved. This is the situation in Figure 2.

The instrument demonstrated strong psychometric properties. Content validity was excellent, with an average Content Validity Ratio (CVR) of 0.82, exceeding the critical value of 0.62 (19, 20). Reliability was also high: the questionnaire’s internal consistency was excellent (Cronbach’s *α* = 0.917), its test–retest stability over 2 weeks was strong (*r* = 0.85, *p* < 0.001), and the inter-rater reliability for observations was substantial (Kappa = 0.76), confirming scoring objectivity. The details are shown in Table 4.

**Table 4:** Reliability and validity results of the research instrument.

Psychometric property	Metric	Result	Interpretation
Content validity	Content Validity Ratio (CVR)	0.82	Excellent (> 0.62)
Reliability			
Internal consistency	Cronbach’s Alpha	0.917	Excellent (> 0.7)
Stability (Test–Retest)	Pearson’s r	0.85	Excellent (*p* < 0.001)
Inter-rater reliability	Kappa Coefficient	0.76	Substantial (> 0.6)

### 3.1 Overall effectiveness evaluation

**Figure 3:**
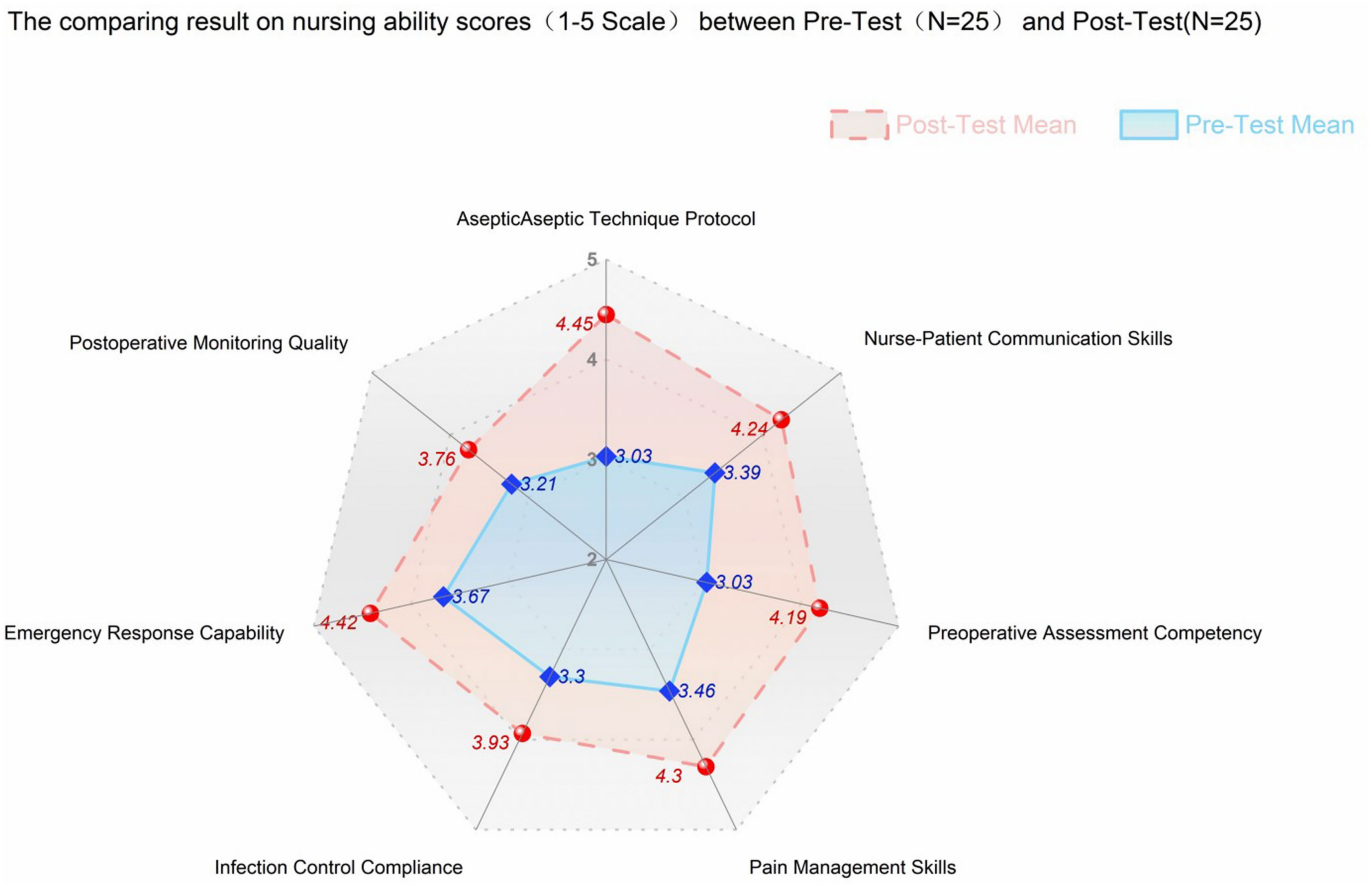
Radar chart comparing nursing ability between pre-test and post-test.

Through a comprehensive assessment, the competency of nurses in handling aseptic technique protocols, emergency response skills, preoperative assessment, pain management, infection control compliance, and nurse–patient communication skills was evaluated using a five-point scale. The findings of the comparison between the pre-training and post-training periods are presented in Table 5, and the improvement in each competency parameter is illustrated in Figure 3.

**Table 5:** Comparison of results of nursing ability scores (N = 25).

Competency	Pre (M ± SD)	Post (M ± SD)	Mean difference	SD difference	*t*	*p*	95%CI
Aseptic technique protocol	3.03 ± 0.37	4.45 ± 0.25	+1.42	0.31	22.90	<0.001	1.42 (1.29, 1.55)
Emergency response skills	3.67 ± 0.28	4.42 ± 0.17	+0.75	0.23	16.30	<0.001	0.75 (0.66, 0.84)
Preoperative assessment skills	3.03 ± 0.45	4.19 ± 0.29	+1.16	0.38	15.26	<0.001	1.16 (1.00, 1.31)
Pain management skills	3.46 ± 0.34	4.30 ± 0.23	+0.84	0.29	14.48	<0.001	0.84 (0.73, 0.95)
Infection control compliance	3.30 ± 0.50	3.93 ± 0.27	+0.63	0.42	7.5	<0.001	0.63 (0.46, 0.80)
Nurse–patient communication skills	3.39 ± 0.37	4.24 ± 0.30	+0.85	0.34	12.5	<0.001	0.85 (0.71, 0.99)
Postoperative monitoring quality	3.21 ± 0.40	3.76 ± 0.35	+0.55	0.38	7.24	<0.001	0.55 (0.39, 0.71)

**Figure 4:**
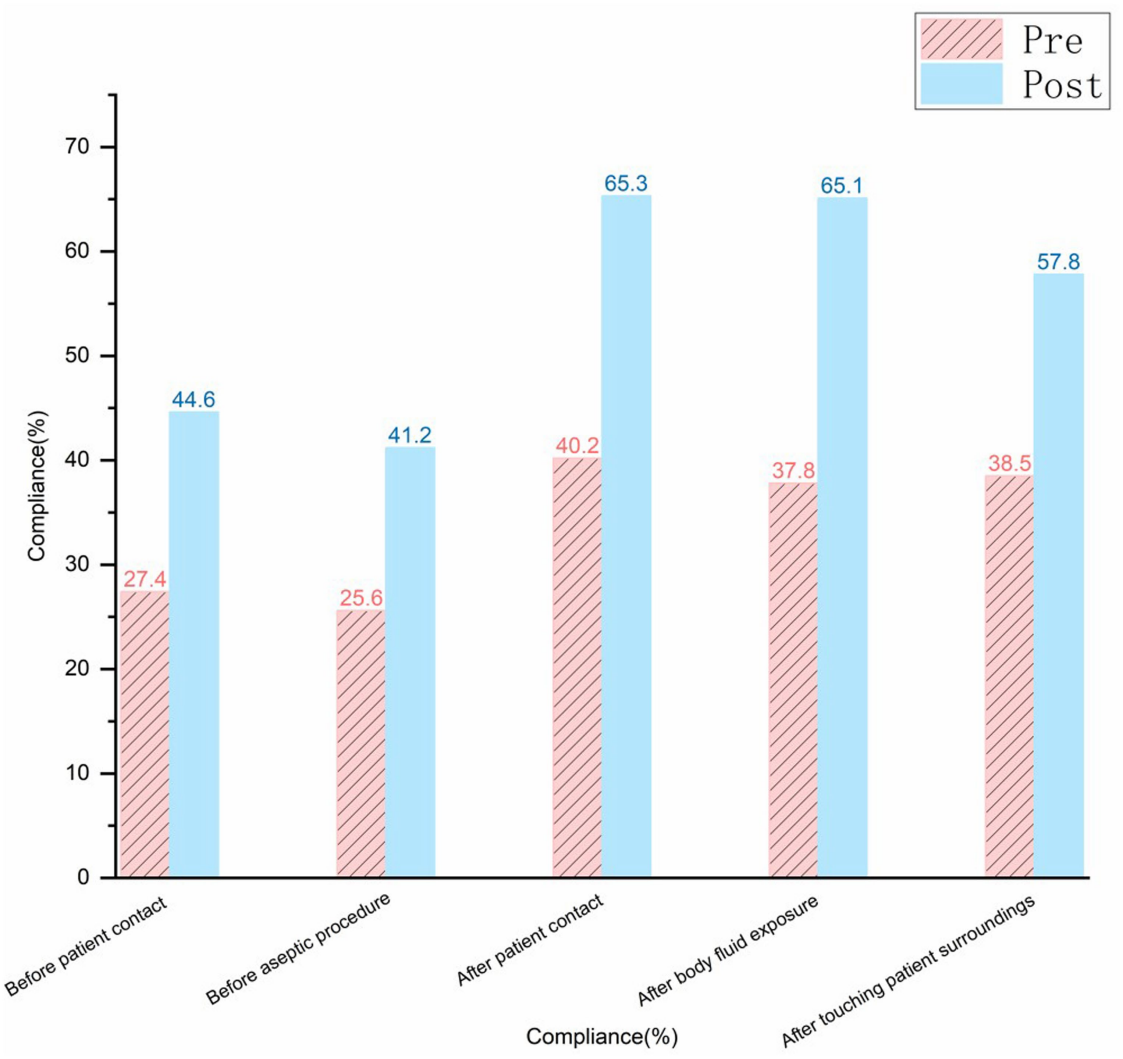
Bar graph comparing Pre and Post compliance percentages in different scenarios.

**Figure 5:**
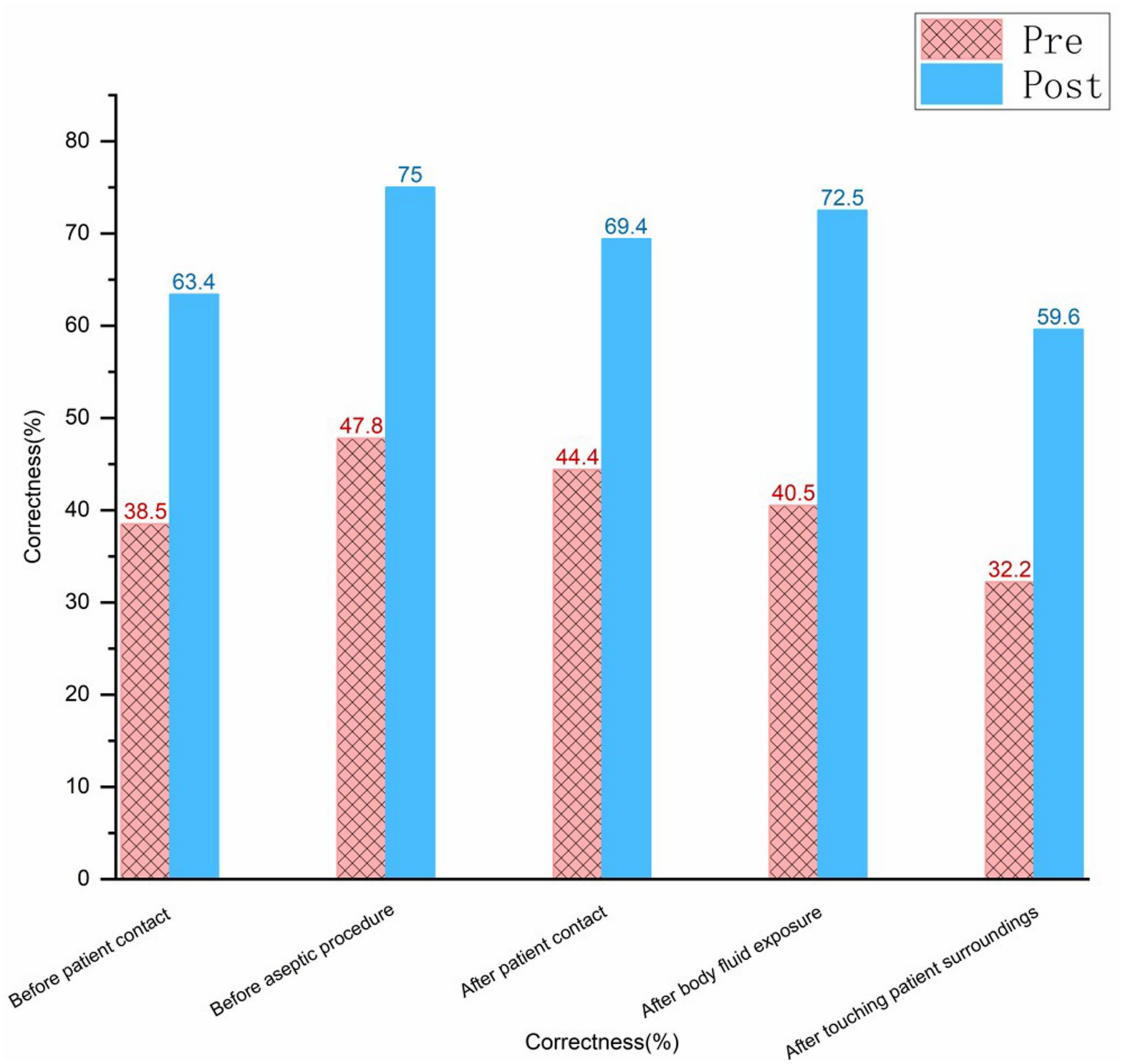
Bar chart comparing correctness percentages before and after intervention across five scenarios.

**Figure 6:**
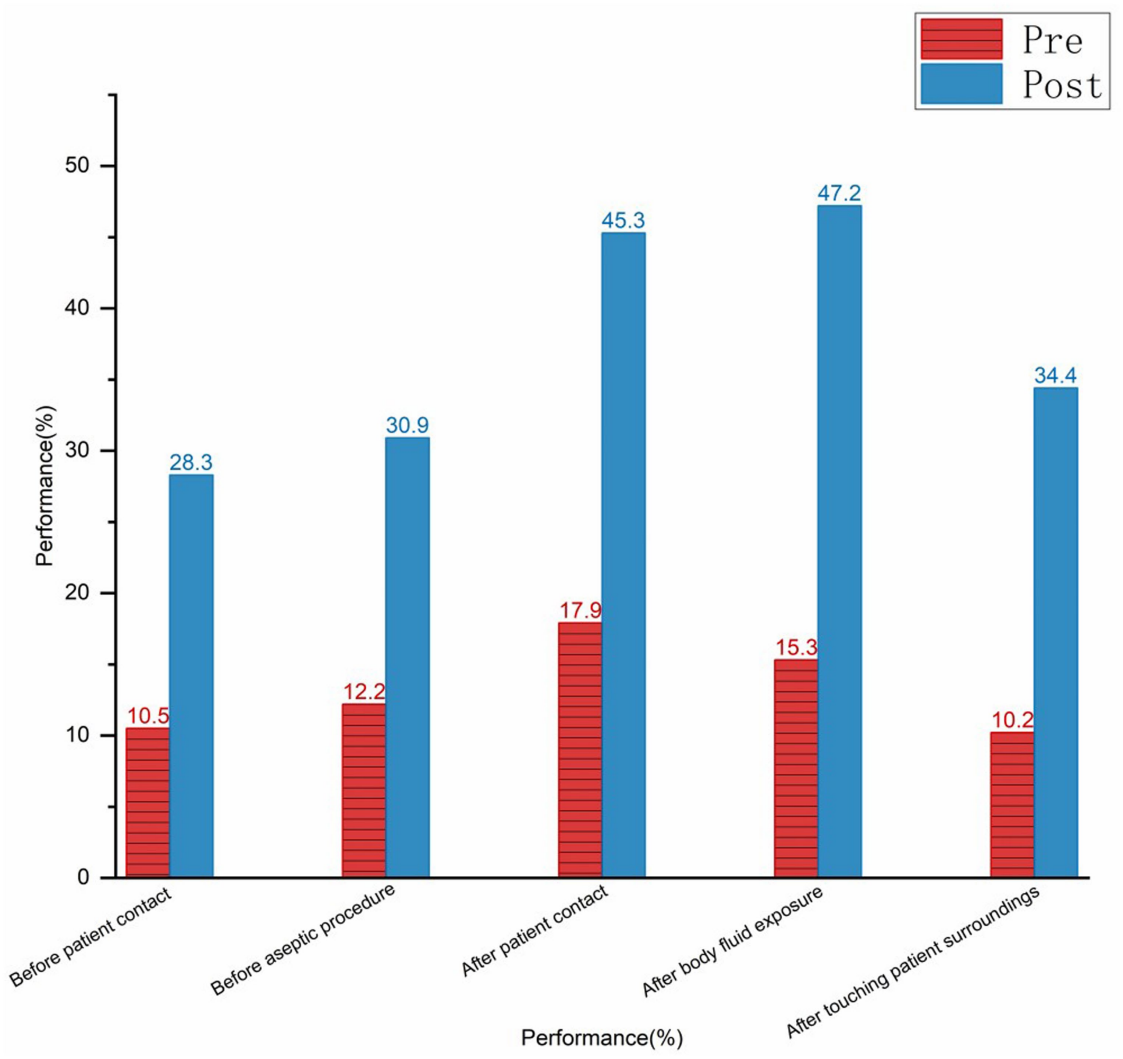
Bar chart comparing performance percentages in hygiene practices before and after intervention.

Hand hygiene timing was observed 970 times before and after the cumulative period. The hand hygiene compliance rate before and after the training improved from 32.6% (162/497) to 55.0% (264/480), and the correct hand hygiene rate increased from 40.6% (202/497) to 60.6% (291/480). The hand hygiene performance at different timings had also improved. The specifics of the various timings are illustrated in Figures 4–6 (compliance rate; correct rate; and performance rate).

Four basic surgical procedures were implemented through a standard operating procedures process assessment, including changes to wound dressing (wound management), aseptic techniques, catheterization, and cardiopulmonary resuscitation. Some operations were implemented through situational simulations, which included explanations of precautions, safety reviews, and hand hygiene. Others were implemented through workshops, focusing on the use of electrosurgical equipment and positioning techniques. The paired t-test results comparing the nurses’ pre- and post-training assessment scores revealed a statistically significant difference in the scores for all four operational skills. The assessment scores for the four basic procedures are presented in Table 6.

**Table 6:** Performance in clinical procedures before and after training (*N* = 25).

Variable	Pre-test Mean ± SD	Post-test Mean ± SD	*t*	*p*
Wound management	87.02 ± 2.11	95.18 ± 1.51	25.943	<0.001
Aseptic	85.40 ± 3.06	93.72 ± 2.07	25.193	<0.001
Indwelling urinary catheterization	88.56 ± 1.89	95.76 ± 1.42	27.213	<0.001
Cardiopulmonary resuscitation	87.00 ± 1.91	95.92 ± 1.29	28.729	<0.001

## 4 Discussion

### 4.1 Quality management in perioperative care faces several challenges

This study examined the current state of perioperative nursing management in hospitals in Lao PDR, developed a strategy to enhance the quality of perioperative nursing care, and assessed its effectiveness. To the best of our knowledge, this is the first study in the Lao PDR to aim at improving the quality of perioperative care by training healthcare providers.

Scientific and systematic training is essential for improving nursing standards, ensuring patient safety and enhancing the quality of healthcare. In the complex and high-risk field of perioperative care in particular, the professional competence of nursing staff has an impact on surgical success and patient recovery (19). According to preliminary feedback from Chinese aid experts, improving perioperative nursing quality at Laos 103 Hospital requires addressing three key issues. The first was the lack of resources and their uneven distribution. The entire hospital’s surgical hand disinfection supplies were inadequate, and the use of irregular infrastructure had not been perfected.

The second was the lack of talent. While providing support to the hospital, most nursing staff had high school or technical secondary education, and an undergraduate degree accounted for little significance. They mainly relied on experience to operate, and the overall talent training route lacked precise planning. In all, the nursing professionals in hospitals in Laos lacked higher education training and professional expertise in providing perioperative care.

Third, a significant challenge to perioperative quality and safety was the absence of a health information system, which necessitated reliance on paper-based documentation. This approach compromised data integrity, evidenced by high rates of under-reporting for serious complications and adverse events. Furthermore, it led to incomplete follow-up, particularly for patients after major surgery, who missed crucial postoperative assessments. To address these systemic deficiencies, we recommend a dual-pronged strategy, on the one hand, developing technological infrastructure through international collaboration to establish electronic monitoring and reporting systems; on the other hand, investing in human capital by sending Laotian nursing scholars to neighboring countries for advanced training in perioperative care, health informatics, and quality improvement.

### 4.2 A strategic framework for enhancing perioperative care quality in Lao PDR

This training enhanced the overall competence of medical and nursing staff in both basic and specialized care, as well as their ability to manage and monitor all aspects of their daily work in hospitals in Laos. Training on surgical safety checklists, intraoperative first aid, risk assessment, and complication prevention fills the gap in local training.

The most fundamental objective of establishing a scientific nursing training system is to systematically enhance the individual clinical competence of nursing personnel. The STEPCARE training model, developed by our research team, was established based on this very objective. Through 4 years of preliminary experience, our nursing team identified the training challenges faced by the operating room nursing team in Laos. Evidence-Based Practice (EBP) provides the theoretical framework for this process, ensuring that nurses master the latest techniques and knowledge (20, 21). Simulation training, in turn, translates this theory into practice. Its effectiveness has been demonstrated in studies ranging from enhancing the hand hygiene capabilities of operation room department nurses to improving the nursing process and patient safety awareness of registered nurses through virtual simulation (22). At the core of these competencies is patient safety, cultivated through specialized educational programs that foster nurses’ ability to identify, assess, and manage risks (23, 24). This focus extends to critical practice areas such as infection control (25) and even advanced cognitive training (26).

However, excellence in perioperative care also relies on efficient teamwork and optimized system processes. To institutionalize this collaborative spirit, systematic quality improvement tools, such as Clinical Pathways (CNP) combined with the PDCA (Plan-Do-Check-Act) cycle, have demonstrated significant value in enhancing the quality of perioperative care (27). Innovative educational models are instrumental in elevating training quality. For instance, a customized blended-learning “Fundamentals of Perioperative Nursing Course” was designed for novice nurses to facilitate a smooth transition into the complex clinical environment (28). This indicates that a training system must be dynamic and adaptable, continuously adjusting its content and format based on clinical feedback and practical needs.

In conclusion, the scientific nursing training framework developed in this study is not merely an accumulation of skills, but rather a holistic quality improvement paradigm that progresses from individual competence to teamwork and finally to system optimization. By empowering individual nurses, strengthening team efficacy, and ensuring a seamless integration of theory and practice, it ultimately lays the foundation for improving perioperative care quality, ensuring patient safety, and enhancing the overall quality of nursing care within the healthcare system (29).

### 4.3 Strategies based on the knowledge, attitude, and practice model to promote the quality of training

This study analyzed the issues related to local nursing talent management from the perspective of an aid worker. It localized advanced perioperative theories, promoted their clinical application, emphasized “localized transformation,” and proposed a strategy that took into account international norms and the actual resources in Lao PDR, thus overcoming the limitations of “fetishism.” The strategic proposition presented in this study aligns with the national policy of Lao PDR, which highlights the need for effective communication and the development of better human resources to address local challenges and barriers (30).

Moreover, existing international research on medical aid tends to focus on resource inputs and less systematically explores how international standards can be adapted to the specific contexts in low-income countries. Regarding methodology, the study adopts the KAP model as its theoretical foundation. It integrates it with in-depth process management, thereby expanding the scope of the traditional KAP model in public health. By integrating it with perioperative process quality management tools (e.g., the Plan-Do-Check-Act cycle), this study forms a closed loop of “knowledge–attitude–practice–quality monitoring” to enhance critical thinking and problem-solving skills, and subsequently promotes systematic behavioral changes. Owing to this disruptive approach, this study improves critical thinking and problem-solving skills and systematically promotes behavioral changes. The research team has innovatively proposed the STEP-CARE model, which enables systematic training, team empowerment, resource adaptation, and practice standardization to form a comprehensive chain of “people–equipment–processes–culture,” ensuring multidimensional collaborative improvement.

In addition, scenario simulation has been used as a teaching method to improve patient safety and quality of care (31), safety reviews, hand hygiene, and other activities. For example, four nurses were selected to play the roles of the doctor in charge, anesthetist, visiting nurse, and hand-washing nurse. Scenarios were then simulated to cover different aspects of safety verification. Another example is our simulation of healthcare workers failing to practice hand hygiene when interacting with various patients or scenarios. We used fluorescent staining to educate patients and healthcare workers about the importance of avoiding contact transmission. The integrated implementation of multiple assessment methods, including standard operating procedures, workshops, and continuous supervision, has led to ongoing improvements in the quality of care. In terms of clinical application, this study focuses on the gaps in perioperative care management in Lao PDR, fills the gaps in empirical data in the region, and provides a reference for the development of national health policies. Combined with qualitative research, policy development, and practice promotion, it ensures that the Plan-Do-Check-Act cycle contributes to improving training quality. The contributions of this study have implications in global health policy and respond to the WHO’s urgent call to reduce surgical mortality in low- and middle-income countries.

Despite its impact and contributions, this study has limitations. It solely focused on the largest hospital in the Lao PDR, and its investigation of other hospitals was minimal and could not be supported by further data. This is a status quo study for which further data could not be obtained. Moreover, this study had a small sample size. The application and effectiveness of the findings should be verified when extrapolating the data to other settings within the country. After further evaluation and improvement, future research can extend the findings to a larger scale in grassroots hospitals in the Lao PDR.

## 5 Limitations

This study has several limitations that warrant careful consideration. First, the absence of a control group restricts our ability to establish causality, as the observed improvements in nursing performance may be influenced by external factors or a natural maturation effect rather than the training program alone. Second, the findings are potentially subject to the Hawthorne effect, where the nurses’ awareness of being monitored could have independently enhanced their performance, thereby inflating the intervention’s measured impact. Finally, while our custom-designed performance checklist underwent preliminary validation, its psychometric properties have not been fully established, which may affect the precision and generalizability of our measurements. Future research should therefore employ a randomized controlled trial and more samples with formally validated instruments to confirm these findings.

## 6 Conclusion

Enhancing the quality of perioperative care in the Lao PDR requires a multifaceted strategy that adapts international guidelines to the local context, strengthens primary care capacity, and establishes a sustainable resource network. For international assistance expert, this strategy should be executed through a dual-track approach: prioritizing quick wins to immediately reduce harm, while simultaneously investing in long-term systemic reforms for lasting impact. The “quick wins” track should focus on deploying high-impact, low-cost interventions, such as promoting hand hygiene, establishing surgical safety verification protocols. In parallel, the long-term reform track must cultivate sustainable improvements by training nursing professionals, developing robust hospital infection surveillance and adverse event reporting systems. By systematically addressing both immediate risks and underlying system weaknesses, this integrated approach will directly reduce perioperative complications, including infections, bleeding, and nurse-related incidents, thereby significantly advancing patient safety and the overall quality of care.

## Data Availability

The raw data supporting the conclusions of this article will be made available by the authors, without undue reservation.

## References

[1] Lekens ALB Drageset S Hansen BS. Knowing how, arguing why: nurse anaesthetists' experiences of nursing when caring for the surgical patient. BMC Nurs. (2025) 24:144. doi: 10.1186/s12912-025-02752-3, 39920699 PMC11803927

[2] De Vries EN Ramrattan MA Smorenburg SM Gouma DJ Boermeester MA. The incidence and nature of in-hospital adverse events: a systematic review. Qual Saf Health Care. (2008) 17:216–23. doi: 10.1136/qshc.2007.023622, 18519629 PMC2569153

[3] Kim NY Jeong SY. Perioperative patient safety management activities: a modified theory of planned behavior. PLoS One. (2021) 16:e0252648. doi: 10.1371/journal.pone.0252648, 34170919 PMC8232430

[4] Chen J Song C Guo X. Effects of seamless care in the perioperative management of laparoscopic pancreatoduodenectomy on patients’ quality of life and postoperative complications. Sci Rep. (2025) 15:8726. doi: 10.1038/s41598-025-92871-3, 40082622 PMC11906768

[5] Gezer D Şişman H Yurtseven Ş. Knowledge and practices of surgical nurses in perioperative hypothermia management: implications for surgical patient safety and outcomes. Appl Nurs Res. (2026) 87:152027. doi: 10.1016/j.apnr.2025.152027 41578981

[6] World Health Organization. WHO guidelines for safe surgery. Geneva: World Health Organization (2009).

[7] Mariee AA Ahmed AF Altarawneh TM Aljohani HA Al-Otaibi MB. The role of operation room’s nursing interventions on surgical site infection and patient outcomes: a scoping review. Haya: Saudi J Life Sci. (2025) 10:157–69. doi: 10.36348/sjls.2025.v10i05.002

[8] Weerakkody RA Cheshire NJ Riga C Lear R Hamady MS Moorthy K . Surgical technology and operating-room safety failures: a systematic review of quantitative studies. BMJ Qual Saf. (2013) 22:710–8. doi: 10.1136/bmjqs-2012-001778, 23886892

[9] Haynes AB Weiser TG Berry WR Lipsitz SR Breizat AHS Dellinger EP . A surgical safety checklist to reduce morbidity and mortality in a global population. N Engl J Med. (2009) 360:491–9. doi: 10.1056/NEJMsa0810119, 19144931

[10] Kaur M Sophia Kaur R. A quality improvement initiative to reduce central line associated blood stream infection in PICU of a tertiary care hospital in North India. Indian J Med Microbiol. (2025) 57:100922. doi: 10.1016/j.ijmmb.2025.100922 40651580

[11] Westman M Takala R Rahi M Ikonen TS. The need for surgical safety checklists in neurosurgery now and in the future–a systematic review. World Neurosurg. (2020) 134:614–628.e3. doi: 10.1016/j.wneu.2019.09.140, 31589982

[12] Rusnell L Nelson G. Future directions in enhanced recovery after surgery (ERAS) for gynecologic surgery. Clin Obstet Gynecol. (2025) 68:538–43. doi: 10.1097/GRF.0000000000000967, 40874438

[13] Storesund A Haugen AS Wæhle HV Mahesparan R Boermeester MA Nortvedt MW . Validation of a Norwegian version of surgical patient safety system (SurPASS) in combination with the world health organizations’ surgical safety checklist (WHO SSC). BMJ Open Qual. (2019) 8:e000488. doi: 10.1136/bmjoq-2018-000488, 30687799 PMC6327875

[14] Fan QQ Feng XQ Jin JF. Nursing rounds: a quality improvement project to improve outpatient satisfaction. J Nurs Manag. (2020) 29:177–85. doi: 10.1111/jonm.13131, 32780532

[15] Zhou M Qi H. Evaluation of continuous improvement effect of perioperative nursing quality in Da Vinci robot-assisted laparoscopic radical prostatectomy. Medicine. (2025) 104:e42896. doi: 10.1097/md.0000000000042896, 40527769 PMC12173331

[16] Sirisomboon R Nuampa S Leetheeragul J Sudphet M Pimol K Sirithepmontree S . Enhancing the competencies of obstetrical nurses and midwives in high-risk pregnancy management through simulation-based training in Lao people’s Democratic Republic: a pilot study. Midwifery. (2024) 137:104132. doi: 10.1016/j.midw.2024.104132, 39111124

[17] Duangmixay S Virachith S Hübschen JM Siphanthong P Suthepmany S Sayasone S . Latent tuberculosis prevalence in healthcare workers in Laos: a cross-sectional study. Trop Med Health. (2025) 53:1. doi: 10.1186/s41182-024-00677-2, 39754226 PMC11697743

[18] Lai D Zhong J Lu T Pu JM. Patient education in sudden sensorineural hearing loss: knowledge, attitude/belief, and practice findings among otolaryngologists and otologists in China. Patient Educ Couns. (2019) 102:93–8. doi: 10.1016/j.pec.2018.08.022, 30146406

[19] Fatimah AW Kamaruddin MKA Lukman ZM Zukri TM Norashida SR Azizah I. Content validity of questionnaire on the influence of housing affordability factors on the well-being of the B40 group using the content validity ratio (CVR). IJRISS. (2023) VII:898–908. doi: 10.47772/ijriss.2023.701070

[20] Singh S Kumar P Priyanka P Masina S Pandey SD Halemani K. Comparison of Biportal and Uniportal Spine Endoscopy for Effectiveness and Perioperative Safety: A Systematic Review and Meta-Analysis. Cureus. (2025) 17:e98764. doi: 10.7759/cureus.98764 41523539 PMC12779580

[21] Elhabashy S Moriyama M Mahmoud EIE-D Eysa B. Effect of evidence-based nursing practices training programme on the competency of nurses caring for mechanically ventilated patients: a randomised controlled trial. BMC Nurs. (2024) 23:225. doi: 10.1186/s12912-024-01869-1, 38566049 PMC10986015

[22] Koy V Preechawong S Yunibhand J Rauth A Bircher N Prak M . Evaluation of nursing process competencies, nursing quality, and patient safety using virtual simulation with debriefing: a quasi-experimental study. Heliyon. (2023) 9:e20341. doi: 10.1016/j.heliyon.2023.e20341, 37767492 PMC10520815

[23] Carvajal Villalba C Tuay Sigua RN Medina Moya JL. Elevating patient safety education in undergraduate nursing: an integrative review beyond trends. Int J Nurs Sci. (2025). doi: 10.1016/j.ijnss.2025.10.009 PMC13424398 42521393

[24] Xu J Chen X Zeng Y Tang J Long Y Li L. Mapping patient safety competency in undergraduate nursing interns: insights from a latent profile analysis. Nurse Educ Today. (2025) 153:106813. doi: 10.1016/j.nedt.2025.106813, 40561893

[25] Lee S-H Choi J-S. Development and implementation of a mobile-integrated simulation for COVID-19 nursing practice: a randomized controlled pretest–posttest experimental design. Health. (2024) 12:419. doi: 10.3390/healthcare12040419, 38391795 PMC10887982

[26] O’Connell KM Garbark RL Nader KC. Cognitive rehearsal training to prevent lateral violence in a military medical facility. J Perianesth Nurs. (2019) 34:645–653.e1. doi: 10.1016/j.jopan.2018.07.003, 30385099

[27] Zhao L Hu L Li Z Deng F. Plan-do-check-action circulation combined with accelerated rehabilitation nursing under computed tomography in prevention and control of hospital infection in elderly patients undergoing elective orthopedic surgery. Contrast Media Mol Imaging. (2022) 2022:4574730. doi: 10.1155/2022/4574730, 35548404 PMC9061006

[28] Kaldheim HKA Munday J Haddeland K Fossum M. Newly graduated perioperative nurses' experiences of transitioning to clinical practice: a qualitative explorative secondary analysis. J Adv Nurs. (2025) 81:3252–67. doi: 10.1111/jan.16537 39425757 PMC12080083

[29] Kumari R Singh Choudhary A Umar M Chauhan A Priya T. Optimizing specialized nursing education in India for enhanced patient care quality: a nurse-patient centric approach. Int J Res Med Sci. (2025) 13:3785–94. doi: 10.18203/2320-6012.ijrms20252792

[30] Viphonephom P Kounnavong S Reinharz D. The state of decentralization of the healthcare system and nutrition programs in the Lao people’s Democratic Republic: an organizational study. BMC Health Serv Res. (2024) 24:1037. doi: 10.1186/s12913-024-11513-y, 39242512 PMC11380397

[31] Cooper S Seaton P Absalom I Cant R Bogossian F Kelly M . Collectively - the education, simulation and safety (ESS) collaboration. Can scholarship in nursing/midwifery education result in a successful research career? J Adv Nurs. (2018) 74:2703–5. doi: 10.1111/jan.13698, 29733436

